# The Potential of Surface-Immobilized Antimicrobial Peptides for the Enhancement of Orthopaedic Medical Devices: A Review

**DOI:** 10.3390/antibiotics12020211

**Published:** 2023-01-19

**Authors:** Barbara Skerlavaj, Gerard Boix-Lemonche

**Affiliations:** 1 Department of Medicine, University of Udine, Piazzale Kolbe, 4, 33100 Udine, Italy; 2 Center for Eye Research and Innovative Diagnostics, Department of Ophthalmology, Institute of Clinical Medicine, Faculty of Medicine, University of Oslo, 0450 Oslo, Norway

**Keywords:** antimicrobial peptides, membrane-active peptides, surface-immobilized peptides, mode of action, titanium, biofilm inhibition

## Abstract

Due to the well-known phenomenon of antibiotic resistance, there is a constant need for antibiotics with novel mechanisms and different targets respect to those currently in use. In this regard, the antimicrobial peptides (AMPs) seem very promising by virtue of their bactericidal action, based on membrane permeabilization of susceptible microbes. Thanks to this feature, AMPs have a broad activity spectrum, including antibiotic-resistant strains, and microbial biofilms. Additionally, several AMPs display properties that can help tissue regeneration. A possible interesting field of application for AMPs is the development of antimicrobial coatings for implantable medical devices (e.g., orthopaedic prostheses) to prevent device-related infection. In this review, we will take note of the state of the art of AMP-based coatings for orthopaedic prostheses. We will review the most recent studies by focusing on covalently linked AMPs to titanium, their antimicrobial efficacy and plausible mode of action, and cytocompatibility. We will try to extrapolate some general rules for structure–activity (orientation, density) relationships, in order to identify the most suitable physical and chemical features of peptide candidates, and to optimize the coupling strategies to obtain antimicrobial surfaces with improved biological performance.

## 1. Introduction

Antibiotic-resistant bacteria have been designated by the World Health Organization as one of the most serious threats to human health [1], notably in biomaterial implant infections [2]. Several bacterial strains, such as the highly resistant *Staphylococcus aureus* and the emergently resistant *S. epidermidis* [3] or *Pseudomonas aeruginosa* [4,5], have shown an increase in antibiotic resistance. Therefore, measures for preventing bacterial colonization and biofilm formation on implant surfaces are crucial. There are several prophylactic approaches available, including screening and decolonization of methicillin resistant *S. aureus* (MRSA) in carriers, antiseptic skin preparation immediately before surgery, and others [6]. Additional prophylactic approaches would be necessary to design suitable biomaterials resistant to bacterial infection. Currently, diverse strategies for orthopaedic applications [7,8,9] are under study, including anti-adhesive polymers [10,11], super-hydrophobic surfaces [12,13], nano-patterned surfaces [14,15], and the application of hydrogels [16,17]; or which attempt to kill bacteria via inorganic coatings, such as copper ions [18], selenium [19,20], silver nanoparticles [21,22], and Zinc [23,24]; or organic coatings, such as surfaces covalently coated with antibiotics [25,26], chitosan derivatives [27,28], cytokines [29] or enzymes [30], and antimicrobial peptides (AMPs) [31,32].

AMPs represent an untapped reservoir of natural molecules with anti-microbial properties [33,34,35] that are considered the first line of defence against pathogens [36]. Until 2016, over 3000 AMPs have been identified and characterized [37], although the majority are not acceptable as medicines for human therapy in their natural state. Natural AMPs showed to be able to suppress Gram-positive, Gram-negative bacteria, and fungi by disrupting bacterial cell membranes, modulating the immunological response, and regulating inflammation [38,39,40,41]. The AMPs antimicrobial properties, cytocompatibility, molecular structures, and mode of action against microbes have been described in detail in many recent reviews [36,38]. Most of the AMPs have a mode of action based on membrane permeabilization, while several authors suggest that the lipopolysaccharides play an important role in the attraction and attachment of the AMPs to the bacterial cell membrane in Gram-negative bacteria [42]. The ability to permeabilize the bacterial membrane accounts for their broad spectrum activity including antibiotic-resistant clinical isolates [43], efficacy against biofilm-embedded microorganisms, and low level of resistance induction [44,45,46,47]. Despite these excellent properties, only a few AMPs or AMP-derivatives were finally approved by the U.S. Food and Drug Administration [39,48,49]. Due to their properties the development of biomaterials with AMPs anchored to the surface could be an effective approach to avoid bacterial colonization [31,50,51]. Several immobilization strategies showed promising results, including the covalent binding of antimicrobial molecules onto the biomaterial surface [2,52,53,54]. However, it is still required to better understand the mode of action of candidate AMPs in the immobilized state to further improve AMP-functionalized biomaterials. Several authors observed that the AMPs activity could vary depending on the orientation of the anchored AMPs or the length of the spacer used to anchor them [31,50,55,56,57,58,59].

This review will provide an overview of current knowledge of the AMP-based coatings for orthopaedic prostheses with an emphasis on their antimicrobial efficacy, probable mode of action, and cytocompatibility. The review will attempt to extrapolate some general rules for structure–activity (orientation, density) relationships. The review delves deeper the most promising coupling strategies to prosthetic surfaces in order to improve the design of modified AMPs coatings with strong antimicrobial efficacy as well as better biological performance.

## 2. Overview of AMPs Covalently Immobilized on a Metal Surface

The studies performed with the aim of developing antimicrobial coatings for orthopaedic prostheses encompass two different approaches: covalent binding of AMPs to the metal surface (titanium (Ti) in most cases), and non-covalent immobilization on implant surface with subsequent controlled release of AMPs in the microenvironment surrounding the implant. These topics have been described more in detail in several recent reviews [53,54,60,61,62,63,64]. In the present review, we will focus on the covalent approach for potential applications in orthopaedics. Several examples of natural AMPs and their synthetic derivatives, covalently bound to the metal surface (Ti in most cases), which demonstrated efficacy in the immobilized state against Gram-positive and Gram-negative pathogens, are displayed in Table 1. The AMPs are grouped into well-known AMP families starting from the mammalian species. AMPs belonging to the cathelicidin family are most represented, followed by those of the histatin family, and peptides which are fragments derived from human proteins (e.g., hLF1-11). There are also several reports on amphibian and insect AMPs, as well as on some peptides from bacterial origin. The latter ones are non-ribosomally synthesized peptides with peculiar structural features (e.g., cyclic peptides, peptides containing D-amino acids, and lipopeptides). Some of them are of particular interest being the only FDA-approved AMPs for clinical applications (in solution) [39,48,49]. Among the AMPs presented in Table 1, there are few natural sequences, i.e., without modifications except those required for addition of tethering moieties: LL-37, histatin 1, magainins, temporin SHa, and bacterial (lipo)peptides. Conversely, most tethered AMPs are modified sequences derived from or inspired by natural ones. The modified AMPs have shorter amino acid sequences (e.g., FK-16, KR-12, BMAP27(1-18)), with insertion of cationic residues (e.g., GL13K, temporin analogues). Shorter sequences are easier and cheaper to synthesize, what would be advantageous in view of a practical large scale application. On the other hand, increased cationicity is expected to favour interaction with bacteria, even more so for surface-immobilized AMPs. Many studies were performed with peptide libraries designed in silico with the purpose to obtain AMPs with improved properties (e.g., “Tet” series [58]), and with hybrid peptides (e.g., cecropin-melittin [65], and melittin-protamin [66]), designed in silico in order to increase the therapeutic index (i.e., the ratio between cytotoxic and MIC concentration) of the parental AMPs. To prevent bacterial colonization of implants, it is crucial to inhibit bacterial adhesion and subsequent biofilm formation on implant surfaces. Therefore, the immobilized AMPs were designed in order to meet this requirement. Consequently, their ability to inhibit bacterial adhesion and formation of biofilm are the most investigated properties. The target microorganisms have been selected among pathogens causing orthopaedic infections including prosthetic joint infections such as *S. aureus* [66,67,68,69,70,71,72,73,74,75,76,77,78,79,80,81], or among other ESKAPE pathogens such as *P. aeruginosa* [65,66,70,71,73,79,82], or dental pathogens *Porphyromonas gingivalis*, *Streptococcus gordonii*, *S. sanguinis*, *Lactobacillus salivarius* [83,84,85,86,87,88,89,90] or strains known to form biofilm such as *S. epidermidis* [91,92,93], or food contaminants such as *Listeria ivanovii* [79,94,95,96]. In one investigation, Godoy-Gallardo et al. immobilized the lactoferrin-derived hLF1-11 on polyamide brushes on Ti to evaluate its’ efficacy against a multispecies biofilm of the oral plaque collected from one volunteer [89]. Although all studies are at preclinical level, several in vivo studies have been performed [66,72,73,74,75,76,77] and their outcomes are reported in major detail in Table 4. In almost all cases the immobilized AMPs proved compatible to osteoblast cells or other relevant cell types, and in some instances the AMP was mixed with other (non-antimicrobial) peptides (e.g., RGD-containing sequences) to further improve cytocompatibility [68,74,75,90]. Aspects relative to this topic are reported in more detail in Table 4.

It is important to note that in general the AMPs selected for immobilization were membrane-active in solution. However, it remains to be clarified whether such mode of action is kept on a surface. In this perspective, it would be important to understand how specific parameters affect AMPs’ activity on surface: (i) those related to the nature of every single peptide (amino acid sequence and composition, charge, hydrophobicity, distribution of charged/hydrophobic residues along the sequence); and (ii) those related to coupling strategy, including coupling chemistry, the presence of a spacer, peptide orientation, and peptide density on the surface. The studies addressing these latter aspects are displayed in Table 2, including reports on AMPs immobilized on model surfaces.

**Table 1:** Features of AMPs covalently bound to a Ti- or other metal-based substrate.

AMP Family	AMP (Name/Sequence)	Origin (Structure)	Substrate	Biological Activity	Ref.
**Mammalian cathelicidins and their synthetic derivatives**	**LL-37**: LLGDFFRKSKEKIGKEFKRIVQRIKDFLRNLVPRTES	Human cathelicidin (alfa-helical)	Ti	In vitro antimicrobial activity against *Escherichia coli*	[59]
	**FK-16**: FKRIVQRIKDFLRNLV-NH_2_	Fragment 17–32 of LL-37	Ti	In vitro antimicrobial activity against ESKAPE pathogens	[82]
	**KR-12**: KRIVQRIKDFLR-NH_2_	Fragment 18–29 of LL-37	Ti	In vitro antimicrobial activity against methicillin-susceptible and –resistant *S. epidermidis*	[91]
	**Tet213**: KRWWKWWRRC	Synthetic peptide of the Tet series	Ti-coated silicon wafers	In vitro antimicrobial activity against *P. aeruginosa*;	[71]
	**Tet213 + several analogues of tet series** **Tet20:** KRWRIRVRVIRKC	Synthetic peptides of Tet library	Ti-coated silicon wafers; Ti-wires	In vitro antimicrobial activity against *P. aeruginosa* and *S. aureus*; In vivo *S. aureus* rat infection model;	[73]
	**HHC36 (Tet213)**: KRWWKWWRR	Synthetic peptide of Tet series;	Ti;	HHC36 mixed together with RGD peptide in different proportions; In vitro antimicrobial activity against *E. coli* and *S. aureus*;	[68]
	HHC36-polymer	HHC36 conjugated to a temperature-sensitive polymer;	Ti rods;	In vivo rabbit *S. aureus* infection;	[77]
	HHC36	HHC36;	Ti wafers and rods;	In vitro antimicrobial activity against *E. coli* and *S. aureus*; in vivo rabbit *S. aureus* infection model;	[76]
**Mammalian cathelicidins and their synthetic derivatives**	FP	Fusion peptide: HHC36 + QK angiogenic sequence added to the N-terminus of AMP	Ti wafers and rods;	In vitro antimicrobial activity against *E. coli*, *S. aureus* and MRSA; in vivo rabbit *S. aureus* infection model	[74]
	HHC36 + RGD	HHC36 and RGD peptides mixed in optimized proportions	Ti squares	In vitro antimicrobial activity against *S. aureus*; in vivo rabbit *S. aureus* infection model	[75]
	**BMAP-27(1-18)**: GRFKRFRKKFKKLFKKLS-NH_2_	Fragment 1–18 of BMAP-27 (alfa-helical)	Ti; Ti and agarose resin	In vitro antimicrobial activity against *S. epidermidis*	[92,93]
**Histatin peptides and synthetic derivatives**	**Histatin 1**: DSpHEKRHHGYRRKFHEKHHSHREFPFYGDYGSNYLYDN	Histidin-rich peptide isolated from human parotid secretion [97]	Ti	Antimicrobial activity not investigated; effects on osteoblast-like cells in vitro (adhesion, proliferation and differentiation)	[98]
	**Dhvar5**: LLLFLLKKRKKRKY	Synthetic peptide derived from the active domain (amino acids 11–24) of histatin 5	Ti	In vitro antimicrobial activity against *S. aureus*	[69]
	**JH8194**: KRLFRRWQWRMKKY	Synthetic peptide inspired by histatin and other salivary peptides [99]	Ti	In vitro antimicrobial activity against *P. gingivalis*	[83]
				effects on osteoblast-like cells In vitro	[98]
**Defensin-derived peptides**	**SESB2V**: [(RGRKVVRR)2K]2KK	Synthetic branched AMP inspired by the C-terminal end of HBD3	Ti alloy	In vitro antimicrobial activity against *B. cereus*, *E. coli*, *S. aureus*, *P. aeruginosa*, in vivo rabbit keratitis model	[100,101]
**Fragments and derivatives from human proteins**	**GL13K**: GKIIKLKASLKLL-NH_2_	Synthetic peptide derived from the fragment 141–153 of Parotid secretory protein [102]	Ti	In vitro antimicrobial activity against *P. gingivalis*, *S. gordonii*	[84,85,86]
	**hLF1-11**: GRRRRSVQWCA	Fragment 1–11 of human lactoferrin	Ti	In vitro antimicrobial activity against *S. sanguinis* and *L. salivarius* and multispecies biofilm	[87,88,89]
	hLF1-11 plus RGD sequence	The antimicrobial and the cell-adhesive sequence are tethered to the same anchor	Ti	In vitro antimicrobial activity against *S. aureus* and *S. sanguinis*; improved osteoblast cell adhesion	[90]
**Amphibian AMPs**	**Magainin 1**: GIGKFLHSAGKFGKAFVGEIMKS	Frog skin secretion	Chitosan-coated stainless steel	In vitro antimicrobial activity against *L. ivanovii*	[95]
			gold	In vitro antimicrobial activity against *L. ivanovii*, *E. faecalis* and *S. aureus*	[94]
	**Temporin SHa**: FLSGIVGMLGKLF-NH_2_ and several analogues	Selected silylated derivatives (N-, C-, and in the middle of peptide sequence); several sequence analogues	Ti	In vitro antimicrobial activity against *E. coli* and *S. epidermidis*;	[103]
			Gold	In vitro antimicrobial activity against *L. ivanovii*	[96]
**Synthetic derivatives of insect AMPs**	**CM (cecropin-melittin)**: KWKLFKKIGAVLKVL-NH_2_	Hybrid peptide composed of residues 1–7 of cecropin A and 2–9 of melittin [104]	Gold nanoparticles deposited to glass and Ti	In vitro antimicrobial activity against *E. coli*, *P. aeruginosa*, *K. pneumoniae*, *S. aureus* and *S. haemolyticus*	[65]
**Synthetic derivatives of insect AMPs**	**Melimine (melittin-protamine)**: TLISWIKNKRKQRPRVSRRRRRRGGRRRR	Hybrid peptide composed of residues 15–26 of melittin (from bee venom) and 16–32 of protamine (from salmon sperm)	Ti disks	In vitro antimicrobial activity against *P. aeruginosa* and *S. aureus*; In vivo subcutaneous mouse and rat models of *S. aureus* infection	[66]
**Plant AMPs**	**Plant-derived cyclotides**: a complex mixture of cyclic peptides	Cyclic peptides purified from *Viola philippica Cav*., a chinese medicinal plant	Stainless steel	In vitro antimicrobial activity against *S. aureus*	[105]
**Bacterial (lipo)peptides and synthetic analogues**	**Daptomycin**: *n*-decanoyl-WND-*cy*(TG-Orn-DADGS-MeGlu-Kyn)	Lipopeptide from *S. roseosporus*	Ti alloy	In vitro antimicrobial activity against *S. aureus*	[67,78]
	**Bacitracin**: ICLEI-*cy*(KOrnIFHDD)	Cyclic AMP from *B. subtilis*	Ti alloy	In vitro antimicrobial activity against *S. aureus* and MRSA	[80]
				In vivo rat femur implant-related infection model	[72]
**Bacterial (lipo)peptides and synthetic analogues**	GZ3.163: 4-methylhexanoyl-C-Dab-Dab-Dab-LF-Dab-Dab-L-NH_2_	Analogue of battacin lipopeptide from *P. tianmunesis*	Glass, Silicon, Ti	In vitro antimicrobial activity against *E. coli*, *P. aeruginosa* and *S. aureus*;	[70]
	Gramicidin A Formyl-VGALAVVVWLWLWLWGNHCH_2_CH_2_OH	The major component of Gramicidin D, a mixture of gramicidins A (85%), B and C [106]	Gold-coated glass	In vitro antimicrobial activity against *E. coli*, *L. ivanovi*, *E. faecalis*, *S. aureus* and *C. albicans*	[79]

Notes: Sp: phosphorylated Serine residue; underlined letters indicate D-amino acids; Dab: α,γ-diaminobutyric acid; *cy*: cyclic macrolactone ring; HBD3: human beta-defensin 3.

## 3. AMPs’ Efficacy in the Immobilized Condition

A clearly evident finding that stemmed out from almost all studies was the increase in the effective antibacterial concentration of immobilized versus soluble AMPs, from micromolar to millimolar in several cases [55,58,107,108]. Believing that the reduced activity was due to insufficient peptide density on surface as a consequence of low yield of coupling steps, efforts to increase the surface density of tethered AMPs were undertaken, either by modifying the coupling scheme for the improvement of the initial steps by applying for instance different Ti treatments, or by using different silanization agents [87], or by replacing the silanization with other supports such as coatings with hydrophylic polymers enriched in moieties available for covalent anchoring of AMPs. Examples of such polymeric coatings are the acrylamide-based co-polymer brushes [73,88,109] formed by co-polymerization of *N*,*N*-dimethylacrylamide (DMA) and *N*-(3-aminopropyl) methacrylamide (APMA), at optimized DMA:APMA ratios, leading to a remarkable increase in the surface density of amino groups available for subsequent reactions such as the addition of suitable linkers for peptide conjugation. In this way, it was possible to achieve an about 10-fold increased peptide density (in the order of magnitude of several micrograms/cm^2^) respect to direct conjugation of AMPs to Ti [59,73]. Another interesting example is a PEG-based hydrogel developed by Cole et al., that made it possible to obtain a gel layer of 60 µm thickness highly rich in reactive groups for peptide anchoring [110]. In general, the studies based on the polymer-mediated approach recorded a potent antimicrobial activity, which was positively correlated with peptide density on surface.

Furthermore, a crucial parameter taken into account to explain the reduced antimicrobial efficacy was peptide mobility upon tethering. Researchers were aware that it could be difficult for surface-tethered AMPs to reach the bacterial cytoplasmic membrane, which is masked by the peptidoglycan, and in Gram-negatives by an additional (outer) membrane. Several studies addressed this issue by investigating the interaction of surface-tethered AMPs with artificial membranes (e.g., calcein release from LUVs [55]), or inferred it on the basis of structural studies (e.g., a lipid-induced conformational transition observed by CD spectroscopy [73,109,111]). These reports provide evidence on the effective membrane-perturbing ability of immobilized AMPs, and the topic is worthy of further study considering the supramolecular complexity of the bacterial cell envelope. It is reasonable that for interaction with whole bacteria, tethered AMPs should be either long enough themselves or attached to the surface via a sufficiently long and flexible handle functioning as a spacer. In fact, several studies highlight the requirement for a spacer to observe or to improve the activity of the immobilized AMPs [55,59,65], and many studies applied distinct approaches to include a spacer. The use of PEG of various lenght is frequent [55,56,59,65,76,107], as well as the modification of the peptide sequence with the addition of selected conventional (e.g., Gly or other residues) or unconventional (e.g., 6-aminohexanoic acid) amino acids at various positions [69,75,88,89,92,93,110]. In the copolymer brush approach, there is not a specifically added spacer, as the brush itself functions as handle and spacer [71,73,88,89,109]. A similar consideration applies to the PEG-based hydrogel in the work of Cole et al. [110].

However, in the literature there are also studies performed without any spacer [32,58,96,103,108]. The publication of Haynie et al. was one of the first studies providing convincing evidence that magainin 2 and several amphiphilic analogues, covalently immobilized on a resin support without spacer, exerted bactericidal activity [108]. The authors postulated that, at least in the case of *E. coli*, it was by contact-killing, although based on a not yet clarified mechanism. Interesting insights about this topic came from the study of Hilpert et al., who investigated a large library of short (mainly 12-mers) cationic AMPs tethered to cellulose sheets without spacer [58]. The authors performed a detailed structure–activity relationship (SAR) study by evaluating the length, overall charge, hydrophobicity, distribution of charged and hydrophobic residues along the sequence, and their position with respect to the anchoring point, concluding that there is no direct correlation between AMPs’ activity in solution vs. activity on surface. In this study, the most active surface-tethered AMP proved active against *P. aeruginosa*, *S. aureus,* and *Candida albicans*, causing membrane depolarization of *S. aureus* and strongly altered morphology of *P. aeruginosa*. These microorganisms have very distinct envelopes, thus stimulating a reconsideration of the mode of killing action of surface-immobilized versus soluble AMPs.

There is no consensus about AMP orientation on surface (Table 2). In the already cited study of Haynie et al., the reversed sequence of magainin 2 was inactive, indicating that peptide orientation was crucial for activity [108]. Gabriel et al. observed activity only when LL-37 was linked to PEG through the N-terminus [59], whereas other cathelicidin-derived peptides (e.g., SMAP-29, BMAP-27(1-18)) were more active when anchored through the C-terminus [93,107]. Several authors did not make a direct comparison between the two orientations. For instance, Godoy-Gallardo et al. consistently used hLF1-11 grafted through the N-terminus [87,88,89], whereas Gao et al. used C-terminally oriented AMPs, and both authors observed potent activity [71,73,109]. Bagheri et al. demonstrated with resin-immobilized membrane active AMPs (namely, the bee venom melittin with cationic C-terminus and more hydrophobic/amphypathic N-terminal region, and the model amphypathic peptide KLAL) that antimicrobial activity depended on the structure and mode of insertion of the single AMP into the membrane [56]. For melittin, which inserts into the membrane perpendicularly, the tethering orientation was important, whilst for KLAL, which follows the “carpet-like” model, it wasn’t. The MIC values of KLAL at any orientation did not change substantially, while those of N-oriented melittin increased remarkably, suggesting that the amphypathic N-terminal region plays a crucial role in the case of melittin [56]. The Masurier group investigated the orientation-dependent activity of the amphibian temporin (13 residues, charge +2) by using five analogues silylated at different positions of the sequence for site-specific anchoring, and observed the highest killing (50–60%) of *E. coli* and *S. epidermidis* with the analog grafted exactly in the middle of the peptide sequence [103]. The authors correlated this finding with the proposed “carpet-like” mechanism for this AMP in solution, in which the peptide molecules interact with bacteria in parallel orientation with respect to the bacterial surface [103]. However, in this study the differently oriented analogues did exert some killing, although to a lower extent (20–40%), possibly suggesting alternative, though less effective, bactericidal mechanisms. Interestingly, the same research group demonstrated in a follow-up study that orientation had no influence when a Lys residue was introduced in the temporin sequence to increase its overall charge [96]. The group of Costa et al. reported antimicrobial activity of the histatin-derived Dhvar5 (14 residues, charge +7) only when conjugated through the N-terminus [69]. This finding could be explained by the head-to-tail amphipathicity of this AMP, with hydrophobic N-terminus and highly cationic C-terminus, clearly indicating that the cationic portion should be exposed for interaction with bacteria. Furthermore, concerning cationicity, Cecropin A proved more effective in killing bacteria [110] and more able to bind LTA when immobilized by the C-terminus [111]. It is interesting to note in this latter case that the C-oriented Cecropin A, thus exposing its positive N-terminal region, was more able to bind LTA from *Bacillus subtilis*, which is more anionic, than that of *S. aureus* that is less negatively charged, suggesting that eletrostatic interaction plays a role also for surface-immobilized AMPs. Further support to this view comes from the work of Han et al., who demonstrated by sum frequency generation (SFG) vibrational spectroscopy analysis that the C-oriented immobilized Cecropin P1 was able to selectively interact with lipid vesicles mimicking bacterial (POPG), but not mammalian (POPC: 1-palmitoyl-2-oleoyl-sn-glycero-3-phospho-choline) cell membranes [112]. Interestingly, these authors did not register any interaction between the C-oriented peptide and the hydrophobic tails of a POPG monolayer, suggesting that the electrostatic interaction between the cationic N-terminal region of the peptide and the anionic POPG is relevant for the immobilized Cecropin P1 [112]. Thus, in the case of Dhvar and cecropins we can conclude that cationicity seems more important than amphypaticity, although this does not apply to other AMPs. Rather, it appears that for immobilized AMPs, active orientation is peptide-specific. 

Hence, it is important to elucidate the mode of action of AMPs in solution and upon tethering to a surface. Detailed information on SAR of a given AMP in solution would be useful for its immobilization in order to avoid coupling strategies that would render the killing action less effective or even impossible. The work performed by Costa et al. offers an interesting example [113]. In this study, the authors exploited the Cys^10^ residue of the highly cationic lactoferrin fragment hLF1-11 to bind this AMP to a chitosan layer deposited on gold, with and without a relatively short but flexible spacer. Interestingly, only hLF1-11 bound through the spacer effectively killed *S. aureus* with a comparable potency with respect to the non-functionalized chitosan, whilst the AMP grafted without spacer attracted bacteria to the surface but did not kill them. One possible explanation could be that the coupling scheme masked a residue (namely Cys^10^) that was crucial for activity. Moreover, considering peptide orientation and mobility, the authors suggested a “rigid exposition” of the N-terminal cationic region (being the Cys residue close to the C-terminus) by the directly grafted AMP, leading to an unproductive attraction of bacteria, whereas the presence of the spacer would add the flexibility needed for effective killing. It is worthy of note that in the publications of Godoy-Gallardo et al., hLF1-11 grafted to silanized Ti through a flexible spacer added to its N-terminus proved effective against biofilm made by oral pathogens [87,88,89].

Studies specifically addressing the mode of action of covalently immobilized AMPs are reported in Table 3. Taking into consideration that in some instances it could be difficult to apply methods suitable in solution, information concerning methodological aspects is also provided.

**Table 2:** Parameters influencing AMPs’ activity in the immobilized condition.

AMP (Name)	Coupling Strategy	Peptide Orientation	Peptide Density on Surface	Spacer	Antimicrobial Effect	Ref.
**Magainin 2 and synthetic analogues**	Peptides synthesized with an acid-stable bond on a commercial polyamide resin	C-terminus	Not applicable	no	Contact-killing of *E. coli* and *S. aureus*; the reversed sequence of magainin 2 does not display activity	[108]
**LL-37**	Random (via amino groups) and site-specific (via a Cys residue added to N-terminus) binding to silanized Ti, with and without spacer	Random with/without spacer; N-terminus with/without spacer	0.78–1.47 × 10^−10^ mol/cm^2^ (amino groups detection by sulfo-SDTB method)	PEG of 5400 Da	Killing of *E. coli* observed only with the N-terminally immobilized AMP with spacer (PI uptake), no correlation with peptide density	[59]
**Tet peptides library (122 AMPs)**	SPOT synthesis of peptides on cellulose by using the CAPE linker chemistry; biotin-streptavidin tethering to plastic	C-terminus; N-terminus;	50 and/or 200 nmol/spot	no	>90% inhibition of *P. aeruginosa* by short (9-, 12-, 13-mer) cationic AMPs (luminescence); decreased viability of *P. aeruginosa*, *S. aureus* and *C. albicans* (CFU counts); no direct correlation with aa sequence parameters; positive correlation with peptide density	[58]
**Cationic and amphiphilic model peptides:** **KLAL and MK5E, and acetylated/PEGylated derivatives**	Solid phase synthesis on different PEG bearing resins by Fmoc chemistry, oxime-forming ligation and thioalkylation	C- and N-terminus and side-chain immobilization	0.024–0.133 and 0.15–0.25 µmol/mg depending on resin (not directly applicable to a surface)	PEG of 3000, 400 and 200 Da, depending on resin	Best effect against *B. subtilis* and *E. coli* with longer spacer even at lower density, no influence by AMP orientation	[55]
**Melittin, buforin 2, tritrpticin, and KLAL**	Coupling of AOA modified synthetic peptides to a PEG bearing resin by oxime-forming ligation	C- and N-terminus	0.02–0.147 µmol/mg resin (not directly applicable to a surface)	PEG of 3 kDa	Best effect with membrane-active KLAL and C-oriented melittin	[56]
**Tet213 + several Tet peptides**	AMPs conjugated to acrylamide-based copolymer brushes covalently grafted on Ti	C-terminus	10–14 peptides/nm^2^ (corresponding to 3–6 µg/cm^2^)	Not specifically added (the brush itself functions as handle and spacer)	Most brush-conjugated AMPs showed similar high potency against *P. aeruginosa* and *S. aureus* (luminescence, fluorescence and CFU counts)	[73]
**Tet213**	Tet213 conjugated to acrylamide-based copolymer brushes covalently grafted on Ti; optimization of composition (DMA:APMA ratio) and graft densities	C-terminus	12–15 peptides/nm^2^, positively correlated with polymer chains density at 5:1 DMA:APMA ratio	Not specifically added (the brush itself functions as handle and spacer)	Antimicrobial activity *against P. aeruginosa* in general positively correlated to peptide surface density (luminescence)	[71]
**IDR1010**	AMP of the Tet series tethered to an acrylamide-based polymer brush formed on quartz slides	C-terminus	7.5–16 peptides/nm^2^ (corresponding to 2.5–5.4 µg/cm^2^), depending on DMA:APMA ratio		Not investigated (focus on structural modifications induced by interaction with LUVs)	[109]
**BMAP27 and other AMPs of diverse origin, structure and mode of action**	Comparison of four different coupling chemistries on preactivated reactive surfaces suitable for grafting of amino-compounds	random	Peptide density expressed relative to that obtained by aldehyde mediated coupling (fluorescent epicocconone staining)	no	Decrease in *E. coli* viability observed with NHS and aldehyde coupled BMAP-27, LL-37 and Polymyxin B (membrane depolarization)	[32]
**SMAP-29**	Coupling of –SH containing AMP to paramagnetic beads, suitable for amino-groups, via a maleimide-bearing heterobifunctional linker, and to silanized glass	N- and C-terminus	3–7 × 10^−3^ µmol/cm^2^ (beads) and 1.8–2.5 × 10^−3^ µmol/cm^2^ (glass)	PEG_12_	Differentiated killing of selected G+ and G- strains; in general soluble more active than immobilized and C-oriented more active than N-oriented AMP	[107]
**hLF1-11**	Coupling of –SH containing AMP to I-CH_2_-groups on APTES-silanized Ti and on acrylamide-based copolymer brushes on silanized Ti	N-terminus with/without spacer	0.9 µg/cm^2^, for coupling without brushes and 1.3–1.7 µg/cm^2^ for coupling to polymer brushes	3 units of 6-aminohexanoic acid for coupling to silanized Ti, no spacer added for coupling to copolymer brushes	Adhesion of and biofilm formation by *S. sanguinis* and *L. salivarius* reduced to different extent; antibacterial effect damped after 2 h samples sonication	[88]
**hLF1-11**	Comparison between silver-coated Ti, AMP-functionalized silanized Ti, and AMP conjugated to acrylamide-based copolymer brushes on Ti	N-terminus with/without spacer	Not reported	3 units of 6-aminohexanoic acid for coupling to silanized Ti, no spacer added for coupling to copolymer brushes;	AMP coupled to polymer brushes most effective against oral plaque adhesion; AMP shows overall comparable potency to Ag in long-term (3 weeks) biofilm inhibition	[89]
**Dhvar5**	Coupling of –SH bearing analogues to –SH derivatized chitosan (coated to Ti) via disulfide bridge formation	N- and C-terminus	1.5–2.4 ng/mm^2^ (fluorescence assay)	Aminohexanoic acid, aminobutanoic acid and Gly-Gly-Cys;	N-oriented AMP has activity against *S. aureus* adhesion regardless of spacer type	[69]
**Hybrid cecropin-melittin**	Coupling of –SH containing peptide to a maleimide function on gold nanoparticles coated to glass/Ti	C-terminus	46–110 µg/cm^2^	PEG of 1 kDa	Dose-dependent bactericidal effect; best effect with PEG and higher density	[65]
**Cecropin A**	Coupling of maleimide-modified analogues to –SH groups exposed on a PEG hydrogel	C-terminus and in the middle of the sequence	90–990 µM depending on coating composition (Determined indirectly by quantification of reactive –SH groups); focus on coating thickness and other properties	Four Gly residues	Potent bactericidal activity against *E. coli* exerted by C-oriented analogues with no influence by the presence of spacer and positive correlation with AMP concentration (Live/Dead fluorescence staining)	[110]
**FK-16**	Coupling of –SH containing peptide to a maleimide function on silanized Ti	C-terminus	6 × 10^−10^ mol/cm^2^ (amino groups detection by sulfo-SDTB method)	6-maleimido hexanoic acid	Viability of ESKAPE pathogens inhibited to various extent except *E. cloacae*	[82]
**Temporin SHa**	Coupling of several analogues, bearing a hydroxysilane moiety at the N- or C-terminus or in the middle of the sequence, to silanized Ti	N- and C-terminus and in the middle of the sequence;|	1.3–1.9 peptides/nm^2^	no	Maximum activity (50–60% killing) obtained with the AMP anchored in the middle of its sequence	[103]
	coupling of analogues to SAM on gold	N- and C-terminus			overall equal potency against *L. ivanovii*	[96]
**HHC36 (Tet213)**	HHC36 conjugated (via click-chemistry) to a temperature-sensitive polymer coated to dopaminated Ti	N-terminus	0.64 µg/cm^2^ (QCM analysis)	Not specifically added (the polymer itself functions as handle and spacer)	Temperature-dependent killing of *S. aureus* and *E. coli* due to peculiarity of the polymer	[77]
**HHC36 (Tet213)**	PEGylated HHC36 conjugated (via click-chemistry) to silanized Ti	N-terminus	0.58–0.92 µg/cm^2^ (QCM analysis)	PEG_12_	Dose-dependent decrease in CFU counts of *S. aureus* and *E. coli*, according to gradually increased AMP density	[76]
**HHC36 and RGD peptides mixed in optimized proportions**	Two phases procedure: each peptide separately conjugated via thiol-ene chemistry to silanized Ti to obtain a gradient surface, then dual-peptide functionalization (same coupling chemistry) by using optimized parameters extracted from the gradient surface;	N-terminus	0.16–0.49 (AMP) and 0.035–0.026 (RGD) µg/cm^2^ (fluorescent dye detection with respect to a titration curve)	A short CPAPAP sequence added to N-terminus as handle/spacer	Best combination of antimicrobial activity and biocompatibility achieved at AMP:RGD molar ratio of 5.3:1	[75]

Notes: PEG: polyethylene glycol; Fmoc: fluorenylmethyloxycarbonyl; AOA: aminooxyacetic acid; NHS: N-hydroxysuccinimide; SPOT synthesis: synthesis of peptide library on cellulose sheets according to Frank R, Tetrahedron 1992; CAPE linker: acid-stable ether bond; DMA: N,N-dimethylacrylamide; APMA: N-(3-Aminopropyl) methacrylamide hydrochloride; SAM: self-assembled monolayers; QCM: quartz crystal microbalance; click-chemistry: copper-catalysed azide-alkyne cycloaddition.

## 4. Mode of Action of Surface-Immobilized AMPs

As already underlined, membrane-active AMPs were selected for immobilization in the belief that these could reach their molecular target also when immobilized on a support. In fact, Rapsch et al. demonstrated that only membranolytic AMPs reduced bacteria viability upon tethering on the glass surface [32]. However, the interaction of this class of AMPs with the bacterial cytoplasmic membrane can occur in several different ways [36,39], which can make all the difference when the peptides are immobilized (see above the discussion about peptide orientation). Furthermore, the cytoplasmic membrane is not directly exposed to the outside environment, being surrounded by additional envelope components (see above the discussion concerning the need for a spacer). Neither peptide orientation nor the requirement of a spacer are unequivocal outcomes, as there are membrane-active AMPs with a distinct distribution of cationic and hydrophobic residues along the sequence, which will behave differently when immobilized. There is no doubt that immobilized AMPs were able to interact with model membranes, or isolated bacterial lipid components such as LTA and LPS [57,73,109,111,112,114], and to perturb them [55,56]. There is also no doubt that immobilized peptides induced membrane depolarization in whole bacteria, investigated by potentiometric fluorescent dye analysis, and cytoplasmic membrane permeabilization (PI uptake/ATP release, extrusion of nucleic acids) [57,58,59,114]. In addition, outer and inner membrane permeabilization of *E. coli* was recorded by a chromogenic assay with AMPs immobilized on gold nanoparticles [65,115]. These findings are corroborated also by morphological analysis performed in most cases by SEM showing dramatically altered morphology of treated microorganisms [58,85,90,92,93]. It remains to be established whether such effects are elicited by direct interaction of the immobilized AMPs with bacterial membranes, especially the cytoplasmic one, or whether they are an indirect consequence of the interaction of immobilized AMPs with the more protruding superficial bacterial components, such as the LTA in Gram-positives or the LPS in Gram-negatives. As demonstrated by Yasir et al. with the hybrid AMP melimine and a shorter highly cationic synthetic derivative Mel4, such interaction occurred and triggered downstream events inside the bacterial cell, starting from LPS or LTA binding, depending on the microorganism (*P. aeruginosa* and *S. aureus*, respectively), followed by cytoplasmic membrane depolarization, permeabilization to fluorescent dyes (Sytox green), ATP release, and nucleic acids leakage [57,114]. The observed effects on *P. aeruginosa* were very similar to those recorded in solution [116], but happened with both peptides at much slower kinetics [114]. On *S. aureus*, some effects were similar (LTA binding, membrane depolarization and ATP release), but in this case melimine coating induced nucleic acids release, whereas Mel4 induced release of autolysins [57]. Such different behaviour of the two AMPs against *S. aureus* was already observed in solution [117], but on surface it occurred more slowly. Moreover, membrane depolarization of both microorganisms (i.e., Gram+ and Gram−) displayed sigmoidal kinetics in comparison to the hyperbolic kinetics recorded in solution, similar to what observed by Hilpert et al. with immobilized Tet peptides [58]. The authors of this latter study postulated an electrostatic imbalance of the anionic bacterial surface, due to the contact with highly cationic AMPs, as the starting point of subsequent lethal events. Consistently, they observed similar kinetics of membrane depolarization induced by the ion chelator Ethylenediaminotetraacetic acid (EDTA) on both bacterial species, in support of their hypothesis [58].

**Table 3:** Mode of action of surface-immobilized AMPs in comparison to that in solution.

AMP (Name)	Structural Features in Solution/Methods	Mode of Action in Solution/Methods	Structural Features on Surface/Methods	Mode of Action on Surface/Methods	Ref.
**Magainin 2**	amphipathic alfa-helical (CD, Raman, FTIR, NMR) [118]	Membrane permeabilization [118]	Analogues with no predicted helical conformation are not active; reversed sequence of magainin 2 not active	Contact-killing	[108]
**LL-37**	amphipathic alfa-helical with self-association into oligomeric bundles (CD, NMR) [119]	Transient toroidal pore formation [119]	N-terminally linked AMP, secondary structure not determined	Membrane permeabilization (PI uptake);	[59]
		Permeabilization of OM and IM of *E. coli* ML35p (chromogenic assay)	Peptide C-terminally linked to gold nanoparticles	Permeabilization of OM and IM of *E. coli* ML35p (chromogenic assay)	[115]
**Tet series, a library of synthetic peptides derived from bovine dodecapeptide and indolicidin**	Transition from random coil to β-structure in the presence of liposomes (CD) [120]	Membrane depolarization of *S. aureus* and *E. coli* (potentiometric fluorescent dye) [120]	SAR study (charge, hydrophobic and polar fraction, hydrophobic moment)	Membrane permeabilization (ATP release, SEM) and membrane depolarization (potentiometric fluorescent dye)	[58]
**Cationic and amphiphilic model peptides**	amphipathic α-helical (CD)	Membrane permeabilization (LUVs, calcein release)	Not determined	Membrane permeabilization (LUVs, calcein release)	[55]
**Melittin, buforin 2, tritrpticin, and KLAL**	Melittin amphipathic α-helical (CD) [118]	Gram- OM and IM permeabilization (LUVs, calcein release)	Not determined	Melittin (C-term) and KLAL induce membrane permeabilization (LUVs, calcein release)	[56]
**Tet20**	amphipathic α-helical in the presence of lipid vesicles (CD)	-	Conformational transition in the presence of lipid vesicles (CD with AMP tethered to polymer brush formed on quartz slides)	-	[73]
**IDR1010**	amphipathic α-helical in the presence of lipid vesicles (CD)	-	Conformational transition in the presence of lipid vesicles (CD with AMP tethered to polymer brush formed on quartz slides)	Not investigated	[109]
**GL13K**	β-sheet conformation [121]	Interaction with artificial membranes and formation of holes [121]	Not determined	*S. gordonii* cell wall rupture (SEM of bacteria cultured in drip-flow bioreactor)	[85]
**Cecropin A**	Transition from random to α-helical conformation in the presence of SDS and LTA from *B. subtilis* and *S.aureus* (CD)	Membrane permeabilization [118]	More β-strand content in water and even more a-helix in the presence of SDS, regardless of orientation; transition to a-helix in the presence of LTA dependent on orientation and LTA type (CD of quartz slides-immobilized AMPs)	LTA-binding by the C-oriented AMP higher respect to the N-oriented AMP, and dependent on LTA type (fluorescence assay)	[111]
**Cecropin A**	Transition from random to α-helical conformation in the presence of 50% TFE with quantitative differences among analogues	Membrane permeabilization [118]	Transition from random to α-helical conformation in the presence of 50% TFE with quantitative differences among analogues	Not specifically investigated; remarkably better killing observed with the C-oriented analog	[110]
**Hybrid cecropin-melittin**	amphipathic α-helical (CD)	Permeabilization of OM and IM of *E. coli* ML35p (chromogenic assay)	Not determined	Permeabilization of OM and IM of *E. coli* ML35p (chromogenic assay)	[65]
**hLF1-11 + RGD anchored together**	-	-	-	Clearly altered morphology of *S. aureus* and *S. sanguinis* (SEM)	[90]
**Cecropin P1**	Transition from random to α-helical conformation in the presence of a PG bilayer (SFG)	Electrostatic interaction of AMP with and insertion into the PG bilayer (SFG)	Immobilized AMP on SAM adopts α-helical conformation in water with reduced signal intensity upon addition of POPG vesicles (SFG)	Immobilized AMP interacts with POPG vesicles by changing its orientation or conformation (SFG)	[112]
**Melimine and a synthetic, highly cationic derivative, Mel4**	Melimine adopts helical structure in the presence of 40% TFE [122]	Cell membrane depolarization of *P. aeruginosa* and *S. aureus* (fluorescence potentiometric dye assay) [122]	CD recorded with free and bound Mel4 in the presence of lipid vesicles (anionic and zwitterionic)	*P. aeruginosa* LPS binding, inner membrane perturbation followed by ATP leakage and DNA/RNA release (both AMPs);	[114]
		*S. aureus* LTA binding, membrane depolarization, ATP leakage and DNA/RNA release (melimine), *S. aureus* LTA binding, release of autolysins, membrane depolarization and ATP leakage (Mel4) (LAL, fluorescence, luminescence)		*S. aureus* LTA binding, membrane depolarization, ATP leakage and DNA/RNA release (melimine), *S. aureus* LTA binding, release of autolysins, membrane depolarization and ATP leakage (Mel4) (LAL, fluorescence, luminescence)	[57]
**BMAP-27(1-18)**	amphipathic alfa-helical (CD) [123]	*S. epidermidis* membrane perturbation (fluorescence assay)	Not determined	Altered morphology of *S. epidermidis* (ghost-like cells observed by SEM), membrane perturbation higher by the C-oriented AMP (fluorescence assay)	[92,93]
**hyperbranched polylysine covalently tethered to Ti**	-	-	Not determined	CFU reduction in *S. aureus* and *E. coli*, ROS production and increased expression of oxidative stress-related genes, remarkably altered morphology (CFU counts, fluorescence, qRT-PCR, TEM)	[124]

Notes: PG: phosphatidylglycerol; POPG: 1-Palmitoyl-2-oleoyl-sn-glycero-3-(phospho-rac-(1-glycerol)); SFG: sum frequency generation vibrational spectroscopy; SAM: self-assembled monolayers; LAL: limulus amoebocyte lysate; TFE: trifluoroethanol; SEM: scanning electron microscopy; ROS: reactive oxygen species; TEM: transmission electron microscopy; CD: circular dichroism spectroscopy; FTIR: Fourier transformed infrared spectroscopy; NMR: Nuclear Magnetic Resonance; OM: outer membrane; IM: inner membrane; LUV: large unilamellar vesicles.

## 5. Cytocompatibility and Additional Effects of Surface-Immobilized AMPs

Membrane-active AMPs often display toxic effects towards mammalian cells, albeit usually at much higher concentrations with respect to the antimicrobial [48,125]. In view of their use for the development of antimicrobial implant coatings, the assessment of complete biocompatibility of such coatings towards host cells is mandatory. Moreover, possible stimulatory effects on osteoblast adhesion, proliferation, and differentiation were investigated, by virtue of the reported effects on osteoblasts of some AMPs [38], as such properties would favour implant integration. 

Biocompatibility of AMP-functionalized samples was evaluated in vitro against mammalian erythrocytes and various nucleated cell types, mainly osteoblasts and osteoblast-like cell lines, fibroblasts, and bone marrow-derived mesenchymal stem cells (BMMSCs) from human, mouse, rat, and rabbit (Table 4). It is amazing how a known membranolytic AMP, which was toxic to cells in solution at concentrations only slightly above those antimicrobial, became not toxic at all upon immobilization. In this study, the authors incubated a mixed culture of *E. coli* and the monocytic cell line U937, which grows in suspension, with the immobilized cathelicidin BMAP-27 for 3 days. Results showed selective killing of *E. coli*, whilst the U937 cells were unaffected and proliferated at their normal rate [32]. In most cases, immobilized AMPs proved neutral to blood cells [58,77,82], and compatible to osteoblasts, fibroblasts, and other cell types, which adhered to and effectively spread on the modified Ti substrates [73,76,84,86,87,88,89,91,92,93]. In some cases, AMP-functionalized samples were assayed in bacteria-osteoblasts co-culture experiments. The rationale of these experiments is the consideration that a biomaterial should be resistant to infection but prone to colonization by host cells, what is translated to the “race for the surface” concept [126,127]. In these type of experiments, Ti samples grafted with AMPs were first challenged with a bacterial suspension for a relatively short time (e.g., 2 h), then incubated with the relevant cells for several hours to allow cell attachment, and then processed for confocal microscopy analysis. By using this approach it was possible to analyse cell attachment and spreading on the AMP-modified Ti substrata, and verify their excellent compatibility also upon pre-challenge with bacteria [92,93]. Osseointegration is of paramount importance for a prolonged lifespan of the implant. So, for orthopedic applications the adhesion of osteoblast to the implant surface represents the starting point, followed by proliferation and osteogenic differentiation. These phenomena were investigated by PCR analysis of specific gene expression, and often also by immuno-fluorescence and confocal analysis of osteoblast cells seeded on AMP-functionalized Ti substrates and cultivated for relatively long periods (7, 14, and 21 days) in osteogenic medium, with a positive impact in majority of cases (Table 4) [84,86,87,88,89,91,98].

In some studies Ti was grafted with AMPs and other (non-antimicrobial) peptides, such as the RGD-containing sequences. For example, Hoyos-Nogues et al. obtained a successful multifunctional coating by coupling to Ti a construct where the AMP hLF1-11 and the cell-adhesive sequence were tethered to the same anchor [90] (Table 4). In a previous study, Lin et al. mixed the synthetic AMP HHC36 (named also Tet213) with an RGD peptide in different proportions and coupled them to Ti via an innovative chemical approach, known as “click-chemistry” or, more precisely, copper-catalysed azide-alkyne cycloaddition [68]. The researchers obtained an ideal combination of the two peptides to achieve a perfectly biocompatible Ti surface refractory to bacterial colonization. The Chinese group successfully exploited the “click-chemistry” approach in several follow-up studies with the same AMP (namely, HHC36 alias Tet213) (Table 4). It is noteworthy that “click-chemistry” is a straightforward chemical process that attracted considerable interest from the general audience after the chemists Barry Sharpless and Morten Meldal received the Nobel prize for this discovery, together with Carolyn Bertozzi for developing click reactions inside living cells [128]. The RGD sequence was used by Fang et al. in a recently published two-phases procedure: first, the cell-adhesive (RGD) and the antimicrobial (HHC36) peptides were separately conjugated via thiol–ene chemistry to silanized Ti to obtain a gradient surface; then, dual-peptide functionalization was performed by the same coupling chemistry by using optimized parameters (e. g. peptide density, reaction time, and reactant concentration) extracted from each gradient surface [75]. In this way, the authors obtained uniformly functionalized Ti surfaces with optimized cytocompatibility and antimicrobial efficacy. Moreover, a “fusion peptide”, composed of the “QK angiogenic sequence”, derived from the 17–25 segment of VEGF, and the AMP HHC36, was conjugated via click-chemistry to silanized Ti to obtain functionalized surfaces with improved properties involving angiogenesis- and osteogenesis-related genes [74].

**Table 4:** Cytocompatibility, effects on osteoblasts and in vivo studies.

Tethered AMP	Cell Type (Assay)	Effects	Co-Culture In Vitro (Outcome)	Animal Model (Outcome)	Ref.
**Tet library on cellulose sheet**	Human red blood cells (hemoglobin release)	No hemolytic activity by tethered AMPs	-	-	[58]
**Tet20 on Ti wire and slides**	Human platelet activation (flow cytometry); complement activation (sheep erythrocytes); osteoblast-like MG-63 cells (cell viability by metabolic dye, cell adhesion by cell counts on SEM images);	No platelet and complement activation; no toxicity to MG-63 cells at 5 d and improved cell adhesion at 48 h cell culture	-	Rat subcutaneous infection model with *S. aureus* (85% CFU decrease 7 d after implantation)	[73]
**BMAP27 coupled to a preactivated reactive surface suitable for grafting of amino-compounds**	Monocytic cell line U937 (live-dead staining)	No cytotoxicity after 2 h-incubation	Selective toxicity against bacteria in a mixed culture of U937 cells and *E. coli*	-	[32]
**hLF1-11 tethered to Ti with various strategies;**	Human foreskin fibroblasts (cell quantification by enzymatic colorimetric assay)	No cytotoxicity at 4 h and 1 d incubation; cell proliferation at 4 h, 1 d, 3 d, and 7 d)	-	-	[87,88,89]
**hLF1-11 and RGD tethered to the same anchor on Ti**	Human sarcoma osteogenic SaOS-2 cells (cell quantification as above, cell morphology by immuno-fluorescence, proliferation by metabolic dye and mineralization by staining with Alizarin Red S)	Cell attachment improved at 4 h in the presence of RGD; increased cell proliferation and mineralization at 27 d culture	Osteoblasts-bacteria co-culture (SaOS-2 cells attachment and spreading after 16 h on samples pre-challenged with bacteria (2 h *S. aureus* and *S. sanguinis*)	-	[90]
**Melimine tethered to Ti disks and buttons (mimicking implants)**	-	-	-	Mice and rats subcutaneous *S. aureus* infection model (mice: 1.1 and 1.3 log CFU reduction after 5 d with 10^5^ and 10^7^ inoculum, respectively, and reduced clinical signs of inflammation; 1 log CFU reduction after 7 d with 10^5^ inoculum; rats: 2 and 1.5 log CFU reduction after 5 d with 10^5^ and 10^7^ inoculum, respectively)	[66]
**GL13K conjugated to silanized Ti**	Human gingival fibroblasts and mouse osteoblasts (fluorescence microscopy)	Cell numbers of both lines increased in time (1 d, 3 d, and 5 d)	-	-	[84]
**GL13K conjugated to microgroove Ti**	Human gingival fibroblasts ((immuno-) fluorescent staining, cell viability by metabolic dye, cell morphology by SEM)	Cell adhesion at 2 h, 4 h, and 6 h, and proliferation at 12 h, 24 h, 48 h, and 3 d, 5 d, 7 d improved	-	-	[86]
**KR-12 tethered to Ti**	Human BMMSCs (cell adhesion by fluorescent staining, cell viability by metabolic dye, cell morphology by confocal microscopy and SEM, osteogenic differentiation by ALP activity, collagen secretion, gene expression by qRT-PCR, mineralization by staining with Alizarin Red S)	Cell adhesion at 1 h, 2 h, and 3 h, and proliferation at 1 d, 3 d, and 5 d improved; good spreading morphology; increased ALP activity at 10 d; increased expression of osteogenic markers at 10 d and 14 d;	-	-	[91]
**FK-16 tethered to Ti**	Human red blood cells (hemoglobin release); human epidermal keratinocytes HaCat (cell viability by metabolic dye)	No hemolytic activity by tethered AMPs; no cytotoxicity upon 3 h incubation	-	-	[82]
**Bacitracin immobilized on Ti alloy rods**	-	-	-	Rat femur implant-related *S. aureus* infection model (reduction in bone pathology by micro-CT evaluation, CFU decrease in rods and bone tissue at 3 w after surgery); rat femur implant osseointegration model (improved osseointegration by micro-CT and bone formation by calcein and alizarin red S staining at 12 w after surgery)	[72]
**HHC36 (Tet213) mixed with RGD peptide in different proportions and coupled to Ti via click-chemistry**	Rat bone mesenchimal stem cells (cell viability by metabolic dye)	Cell viability after 24 h decreased at 100% AMP and increased with increasing RGD%	-	-	[68]
**HHC36 conjugated (via click-chemistry) to a temperature-sensitive polymer coated to Ti**	Rabbit red blood cells (hemoglobin release); BMMSCs (cell viability by metabolic dye and cell counts and morphology by confocal microscopy)	No hemolytic activity; improved cell viability and adhesion after 48 h	-	Rabbit *S. aureus* infection (91–99% CFU decrease and good biocompatibility after 7 d implantation)	[77]
**PEGylated HHC36 conjugated (via click-chemistry) to silanized Ti**	Mouse BMMSCs (metabolic dye and confocal microscopy)	Good spreading morphology and negligible cytotoxicity at highest peptide densities after 24 h incubation	-	Same as above (marked CFU decrease and good biocompatibility 7 d after implantation)	[76]
**Fusion peptide: HHC36 with QK angiogenic sequence added at the N-terminus, conjugated via click-chemistry to silanized Ti**	Human endothelial (HUVEC) and bone marrow mesenchymal stem cells (gene expression by qRT-PCR; immunofluorescence; metabolic dye)	Improved cell adhesion, spreading and proliferation (both cell types); in vitro angiogenic and osteogenic activity	-	Same as above with >99% killing after 7 d, reduced inflammation and increased vascularization at 14 d, and vascularization and osseointegration at 60 d; vascularization and osseointegration observed also in a non-infection model	[74]
**HHC36 and RGD peptides, mixed in optimized proportions, coupled to Ti by thiol-ene chemistry**	Mouse BMMSCs (metabolic dye and confocal microscopy)	Better cell adhesion and spreading on gradient surface with higher RGD density observed by microscopy at 24 h, cell viability on optimized Ti substrate determined by metabolic dye at 1 d and 3 d	-	Rabbit *S. aureus* infection model (>99% killing after 7 d and remarkably less inflammatory cells by HE staining; remarkably improved osseointegration by histochemistry at 7 d, 30 d and 60 d)	[75]
**BMAP-27(1-18)**	Osteoblast-like MG-63 cells (cell viability by metabolic dye, cell adhesion and morphology by cell counts on confocal microscopy images)	Optimal adhesion and viability of osteoblasts to Ti substrates after 4 h, without significant difference between N- and C-oriented AMP	Osteoblast-bacteria co-culture (MG-63 + *S. epidermidis*) (Remarkably increased surface coverage at 6 h and 24 h also on bacteria-challenged AMP-samples), no significant difference between N- and C-oriented AMP	-	[92,93]
**Histatin 1 and JH8194 bound to Ti via tresyl chloride-activated technique**	Mouse MC3T3-E1 preosteoblasts (cell morphology, adhesion and proliferation by cell counts, SEM analysis and metabolic dye; osteogenic differentiation by ALP activity and RT-PCR analysis of specific marker expression)	Cell adhesion and proliferation at 3 d and 7 d significantly increased on both AMPs; specific genes expression and ALP activity increased at 7 d and 14 d, but JH8194 was always less effective than histatin 1	-	-	[98]

HE: staining with hematoxylin and eosin; ALP: alkaline phosphatase.

The efficacy of Ti samples, functionalized with selected AMPs, was also tested in vivo in rodent subcutaneous infection and rabbit osteomyelitis models. It is the case of Tet20, melimine, bacitracin, HHC36 [66,72,73,76,77], HHC36 mixed with the cell-adhesive RGD sequence [75], and of the fusion peptide (QK angiogenic sequence + HHC36) [74]. In all these studies, the infection was induced by *S. aureus*, which is the predominant pathogen of prosthetic joint and other orthopedic infections [6,129]. The main outcome was the reduction in CFUs on the infected implant and in the surrounding tissues, as well as the reduction in clinical signs of inflammation, at 7 days after implantation and infection (Table 4). In addition, improved osseointegration was observed by histochemical analysis of the sampled tissues in the rabbit model at 7, 30, and 60 days [75], and at 14 and 60 days [74]. Importantly, in this latter study the osteogenic process was monitored also in the absence of infection, with increased vascularization and osseointegration observed at 60 days post-surgery. Improved outcomes concerning inflammation, osseointegration and new bone formation with bacitracin-functionalized Ti rods in the rabbit model were recorded by Nie et al. at 3 weeks after infection, and at 12 weeks after surgery without infection [72].

In the perspective of the development of biomaterials with covalently bound AMPs, there are several issues to be addressed. For instance, the coupling procedures should be simplified as much as possible in order to optimize the overall yield and render the whole process rapid, straightforward, and cost-effective. The adoption of the “click-chemistry” [68] seems promising, as already discussed. Additional fascinating approaches can be found in the literature, such as the use of chimeric peptides with Ti -binding ability, and the use of 3,4-dihydroxy-L-phenylalanine (DOPA)-conjugated peptides with mussel-inspired adhesion ability. In the first case, synthetic cationic AMPs were conjugated to Ti-binding peptides selected by cell surface display and phage display methods, thus obtaining bifunctional chimeric peptides, able to bind Ti and kill Gram-positive and Gram-negative bacteria [130]. In a recent report, the AMP Tet213 was tethered to Ti through its N-terminus by using another Ti -binding sequence, forming a four-branched construct, bound to Ti and linked to the AMP through a flexible spacer [131]. Ti samples decorated with such a construct were cytocompatible and broadly antimicrobial in vitro, and proved able to kill *S. aureus* in a rabbit osteomyelitis model. The nature of the interaction of such peptides with the metal is not considered covalent, but rather dependent on electrostatic interactions. However, in this latter study it demonstrated stability for at least 24 h [131]. 

Similar considerations apply to the DOPA approach, which was inspired by the adhesive properties of the mussel foot proteins, rich in this catecholamine, and attributed to the ability of the catechol moiety to form various, mainly non covalent, interactions with organic and inorganic surfaces [53]. Recently, a synthetic AMP modified with one to seven DOPA residues, added to its C-terminus, was successfully immobilized on titanium by exploiting the adhesive properties of catechol. In fact, in this study the surface density of this AMP was directly correlated to the number of added DOPA residues, with similar increase in the antimicrobial activity. Furthermore, the effectiveness of this approach was confirmed by testing the antimicrobial activity in a rat subcutaneous implant model. It is interesting to observe that although the interaction with Ti surface is not covalent, in this latter study it proved stable at 4 °C and 25 °C for about three months [132]. The DOPA approach was also used by Wang et al. to immobilize DJK-5, a synthetic host defence peptide endowed with immunomodulatory properties, onto titanium alloy with promising outcomes [133].

Another important issue is the stability of the AMP-coated Ti samples, including heat stability, stability towards serum and other biological fluids, and resistance to proteolytic degradation. Heat stability was reported for the hybrid AMP melimine in solution in one experiment where the AMP was autoclaved without losing its bactericidal potency [134]. Using AMPs endowed with such a property would be very advantageous considering that implants should undergo a thorough sterilization procedure before implantation. For in vitro investigations, various Ti samples, functionalized with different AMPs, were sterilized by at least a 30 min treatment with 70% ethanol [69,86,92,93,113], but such a procedure would be acceptable on preclinical level only. Antimicrobial activity of tethered AMPs was negatively affected by the presence of 10–20% human serum [65,82], while the effect of human saliva on GL13K was less deleterious. Chen et al. investigated this problem with the peptide, either covalently bound or physically adsorbed to Ti, by monitoring the release of the fluorescently labelled AMP in human saliva for 11 days [85]. In this experiment, the covalently bound peptide proved remarkably more stable, and thus more suitable for dental applications, with respect to the physisorbed one [85]. High degree of resistance to proteolytic degradation was reported by Wadhwani et al. with model amphiphylic AMPs, conjugated to gold nanoparticles, exposed to trypsin up to 24 h, with full conservation of their antimicrobial efficacy [135]. 

## 6. Conclusions and Future Outlook

In summary, we reviewed the current knowledge on AMPs from various origins, that were successfully tethered to a titanium surface by means of different coupling strategies, and that demonstrated antimicrobial efficacy in the immobilized condition. During the last three decades, the literature on this topic has grown impressively, thus indicating interest by the scientific community for possible orthopaedic applications of AMPs or, more generally, for their applications in the field of implantable medical devices. 

Based on the collected literature, it is possible to deduce some features of the best performing peptide-functionalized metal surfaces. There are essentially three aspects that are crucial and that have been discussed in this review: (i) the density of peptide molecules on the surface, which is positively correlated with antimicrobial efficacy; (ii) the mobility of the grafted peptides, which is not always mandatory, but in most cases required for effective killing action, and (iii) the orientation with respect to the anchoring point, which is the least clear aspect so far. The latter two factors and the third one in particular depend on each peptide’s mode of action. When tested in solution, the peptides selected for tethering were membrane-active, and, as such, endowed with a certain degree of amphipathicity. For membrane-active AMPs in solution, the correlation between their secondary structure/conformational transitions and interaction with target membranes, causing its depolarization/permeabilization, is well characterized. On the contrary, when constrained on the metal surface, these AMPs behave differently and investigating them is complicated by the presence of the metal, which is not suitable, for instance, for the application of CD spectroscopy. Selected AMPs were tethered to model glass surfaces or special resins to elucidate their secondary structure and membrane perturbation ability, respectively. So, deducing sequence- and structure-related parameters for optimal peptide candidates for covalent immobilization is not as straightforward. However, by analysing the performance of selected tethered AMPs, one can deduce that peptide cationicity is an important parameter that enables the surface-constrained AMPs to interact with the negative bacterial surface and elicit downstream effects, leading to bacterial death, although the underlying mechanism has still to be elucidated in its molecular details.

There are additional aspects to be considered in view of the clinical applications of Ti-tethered AMPs, and should be addressed in future studies, such as heat stability, and stability in the biological settings (e.g., in the presence of serum, synovial fluid, proteases). Moreover, the stability of the peptide-functionalized titanium surfaces is particularly important in order to avoid peptide molecules shedding in the surrounding tissues at sub-MIC concentrations, which could lead to the generation of resistant strains. 

At last, but not less important, it would be advantageous to make the coupling procedures as easy and quick as possible in order to obtain cost-effective devices. To that goal, using optimized AMPs with short and simple amino acid sequences would be an additional advantage. Finally, in the light of the more recent in vivo studies demonstrating not just efficacy in infection reduction, but also in stimulating osseointegration of AMP-functionalized titanium samples, surface-immobilized AMPs show potential for orthopaedic medical device enhancement, which is worthy of further investigation.
